# Effect of Evolocumab on Lipoprotein(a) and PCSK9 in Healthy Individuals with Elevated Lipoprotein(a) Level

**DOI:** 10.3390/jcdd7040045

**Published:** 2020-10-15

**Authors:** Olga Afanasieva, Marat V. Ezhov, Elena Klesareva, Oksana Razova, Uliana Chubykina, Mane Egiazaryan, Ekaterina Sherstyuk, Marina Afanasieva, Elena Utkina, Sergei Pokrovsky

**Affiliations:** 1 Institute of Experimental Cardiology, National Medical Research Center of Cardiology, Ministry of Health of the Russian Federation, 121552 Moscow, Russia; afanasieva.cardio@yandex.ru (O.A.); hea@mail.ru (E.K.); razova1@yandex.ru (O.R.); katyushiksher@mail.ru (E.S.); miafanasieva.cardio@yandex.ru (M.A.); utkelena@yandex.ru (E.U.); dr.pokrovsky@mail.ru (S.P.); 2 AL Myasnikov Institute of Clinical Cardiology, National Medical Research Center of Cardiology, Ministry of Health of the Russian Federation, 121552 Moscow, Russia; marat_ezhov@mail.ru (M.V.E.); uliankachubykina@gmail.com (U.C.)

**Keywords:** lipoprotein(a), proprotein convertase subtilisin/kexin type 9, Evolocumab, PCSK9-lipoprotein complex

## Abstract

**Background and aims:** The aim of this study was to investigate the influence of a single injection of Evolocumab on the dynamics of Lp(a), fractions of apoB100-containing lipoproteins, PCSK9, and their complexes in healthy individuals with elevated Lp(a) levels. **Methods:** This open-label, 4-week clinical study involved 10 statin-naive volunteers with Lp(a) >30 mg/dL, LDL-C < 4.9 mmol/L, and a moderate risk of cardiovascular events. The concentrations of Lp(a), lipids, PCSK9, circulating immune complexes (CIC), and plasma complexes of PCSK9 with apoB100-containing lipoproteins (Lp(a)–PCSK9 and LDL–PCSK9) were measured before and each week after Evolocumab (MABs) administration. **Results:** After a single dose injection of 140 mg of MABs, the median concentration of PCSK9 in serum increased from 496 to 3944 ng/mL; however, the entire pool of circulating PCSK9 remained bound with MABs for 2–3 weeks. LDL-C level decreased significantly from 3.36 mmol/L to 2.27 mmol/L during the first two weeks after the injection. Lp(a) concentrations demonstrated multidirectional changes in different patients with the maximal decrease on the second week. There were no positive correlations between the changes in levels of Lp(a), LDL-C, and TC. The change in the amount of circulating complex of PCSK9–Lp(a) was significantly less than of PCSK9–apoB100 (−5% and −47% after 1 week, respectively). **Conclusions:** A single administration of monoclonal antibodies against PCSK9 (Evolocumab) in healthy individuals with hyperlipoproteinemia(a) resulted in a decrease of Lp(a) of 14%, a 5% decrease in PCSK9–Lp(a), a 36% reduction of LDL-C, a 47% decrease in PCSK9–apoB100 and a tenfold increase in total serum PCSK9 concentration.

## 1. Introduction

An increased concentration of lipoprotein(a) [Lp(a)], or hyperlipoproteinemia(a) [hyperLp(a)], is an independent genetic risk factor for the development and progression of atherosclerosis in different vascular beds and subsequent cardiovascular events [1]. An Lp(a) particle consists of an apoB100-containing low-density lipoprotein (LDL)-like particle covalently linked to a highly glycosylated and extremely polymorphic apolipoprotein(a) [apo(a)]. Plasma levels of Lp(a) are genetically controlled and cannot be reduced by statins, while the application of high doses of nicotinic acid has only minor effects on its concentration [2].

Proprotein convertase subtilisin/kexin type 9 (PCSK9) is the key mediator that regulates the recycling of LDL-receptors on the cell surface during the process of binding and internalization of LDL particles. Recent studies have shown that an increased level of PCSK9 is a marker of the presence and severity of atherosclerosis in coronary and peripheral arteries in patients with heterozygous familial hypercholesterolemia [3]. Therapeutic monoclonal antibodies (MABs) that inhibit the activity of PCSK9 initiated a new era in the treatment of severe lipid metabolic disorders [4,5]. Administration of PCSK9 inhibitors enables enhanced recycling of LDL receptors on the surface of hepatocytes and therefore promotes LDL clearance from the bloodstream. Evolocumab and Alirocumab are fully humanized MABs that inhibit PCSK9 and were approved by the FDA in 2015 for treatment of hypercholesterolemia in combination with statins. However, the ability of PCSK9 inhibitors to decrease Lp(a) concentration by 30% [6] was unexpected as the LDL receptor is thought unlikely to contribute to Lp(a) catabolism [7,8]. Despite many studies, the mechanism of this phenomenon remains unclear [9], as does the metabolism of Lp(a) [10].

One of the possible mechanisms of action of these therapeutic MABs is the formation of a circulating triple immune complex of antibodies against PCSK9 with PCSK9-bound Lp(a) [PCSK9-Lp(a)], followed by elimination of this complex from the bloodstream. There are no specific studies on effects of therapeutic MABs on the Lp(a) concentration.

The aim of this study was to investigate the influence of a single injection of Evolocumab on the dynamics of Lp(a), PCSK9, and their complex in healthy volunteers with elevated Lp(a).

## 2. Materials and Methods

We conducted an uncontrolled clinical study to evaluate the influence of single-dose of Evolocumab on the concentrations of Lp(a), fractions of apoB-containing lipoproteins, and PCSK9 in statin-naive subjects with an elevated level of Lp(a). The study was approved by the Ethics Committee of the National Medical Research Center of Cardiology of the Ministry of Health of Russian Federation. 

Patients: The study involved 10 volunteers (7 men, 3 women) with Lp(a) level above 30 mg/dL aged 30–65 years (median [25%; 75%], 44 [36; 57]. All patients had a moderate risk of cardiovascular events (SCORE 1%–5%) according to SCORE chart from 2016 EAS/ESC Guidelines on Dyslipidemia. Patients did not receive lipid-lowering therapy, and had a level of LDL cholesterol (LDL-C) of less than 4.9 mmol/L. Initial screening of individuals was carried out using the database of the Department of Atherosclerosis of the National Medical Research Center of Cardiology, followed by a medical interview, collection of demographic data, and clinical examination in order to determine their risk profile. All subjects gave their informed consent for inclusion before they participated in the study. The study was conducted in accordance with the Declaration of Helsinki, and the protocol was approved by the Ethics Committee in Clinical Cardiology of National Medical Research Center of Cardiology of Ministry of Health of the Russian Federation on 29 January 2018 (Project identification code 233).

The exclusion criteria were:

1. Atherosclerotic cardiovascular disease (ischemic heart disease, stroke, peripheral arterial disease) requiring lipid-lowering therapy;
2. Positive stress test;
3. Chronic inflammatory diseases;
4. Active liver disease;
5. Renal failure (Cockcroft and Gault creatinine clearance of less than 60 mL/min);
6. Type 2 diabetes mellitus;
7. Uncontrolled hypothyroidism (TSH level of 1.5 UNL);
8. Increased level of creatine kinase (CK) of 3 UNL;
9. Immunodeficiency or autoimmune disorders;
10. Administration of any lipid-lowering drugs or supplements affecting the lipid profile;
11. Significant clinical or psychological condition that may interrupt the experiment, including alcohol abuse.

**Figure 1:**
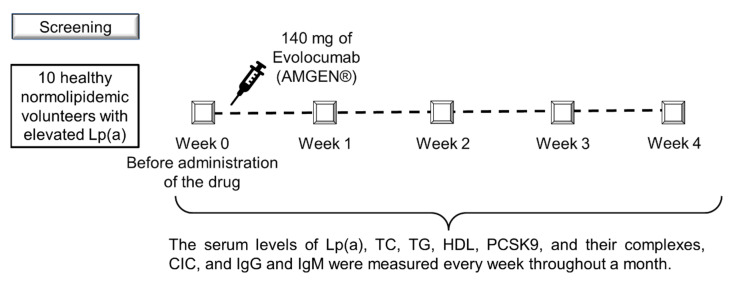
Study design.

The study design is shown in Figure 1.

Laboratory testing: The concentration of Lp(a) in serum samples was measured with an enzyme-linked immunosorbent assay (ELISA) [11]. The levels of total cholesterol (TC), high density lipoprotein cholesterol (HDL-C) and triglycerides (TG) were determined by an enzymatic method Biocon/Analyticon (Germany) using Anthos 2010 microplate spectrophotometer (Anthos, England). The level of LDL-C and LDL-C corrected (LDL-Ccorr) for Lp(a) cholesterol was estimated with the Friedewald formula: LDL-C = TC – HDL-C – TG/2.2 and LDL-Ccorr = LDL-C – 0.3 × Lp(a)/38.7 (mmol/L) [12].

**Figure 2:**
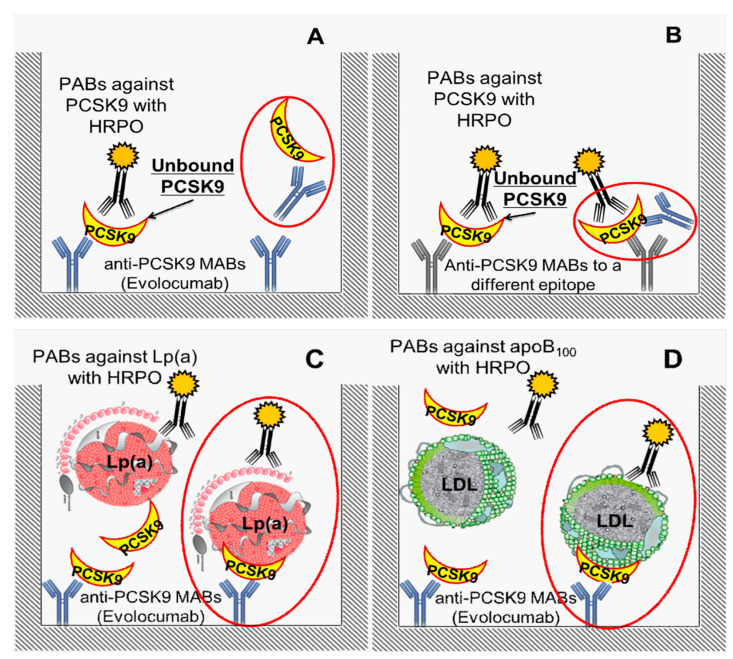
ELISA scheme for measuring: (**A**) PCSK9 not associated with MABs, (**B**) total pool PCSK9 unbound from and bound with MABs. Red circles highlight the complex of PCSK9 with MABs (Evolocumab); (**C**) PCSK9-Lp(a) and (**D**) PCSK9-apoB100 complexes. Red circles are demonstrated the detectable complex of coupled antibodies-antigen-second antibodies conjugated with horseradish peroxidase (HRPO). Abbreviations: PCSK9—proprotein convertase type 9 subtilisin/kexin, MABs—monoclonal antibodies, PABs—polyclonal antibodies, HRPO—horseradish peroxidase, LDL—low density lipoproteins.

The total amount of PCSK9 in serum was determined with a Human Proprotein Convertase 9/PCSK9 kit (R&D Systems, Minneapolis, MN, USA). To estimate free PCSK9 unbound from therapeutic antibodies in serum, the same R&D Systems kit was used with the following modification of the coupled antibodies on the plates: 100 µL of human MABs against PCSK9 with the concentration of 140 mg/dL (Evolocumab, AMGEN, Breda, The Netherlands) were diluted in 10 mM phosphate buffer with pH of 7.4 and coupled in Nunc MaxiSorb plates (Thermo Fisher Scientific Nunc A/S, Roskilde, Denmark) at a final concentration of 100 µg/mL. These plates were used to determine the concentration of PCSK9 unbound from MABs. The calibrator, control serum, horseradish peroxidase (HRPO) conjugated antibodies, and the sequence of the assay itself were completely identical to the assay for determination of the total amount of PCSK9 using the R&D kit (Figure 2A,B).

Circulating immune complexes (CIC) were measured by an immunoturbidimetric assay with reagents kit CIC-Хema (Xema-Medica Co. Ltd, Moscow, Russia) for the determination of circulating immune complexes in human serum or plasma. The test is based on selective polyethylene glycol (PEG 6000, AppliChem GmbH, Darmstadt, Germany) precipitation of CIC in microwells. CIC level is determined by comparison of optical density values in wells with and without PEG at 450 nm. The coefficient of variation of the CIC-Xema Kit does not exceed 8.0%; the recommended upper limit for healthy individuals is 120 lab units.

To determine the concentrations of the complexes of PCSK9 with apoB-containing lipoproteins: Lp(a)–PCSK9 and apoB100–PCSK9 complexes, samples of human MABs against PCSK9 with a concentration of 140 mg/mL (AMGEN, Breda, The Netherlands) were diluted in 10 mM phosphate buffer with pH of 7.4 to a final concentration of 100 µg/mL and ELISA plates Costar® (Corning Incorporated, NY, USA) were incubated for one hour at 37 °C, then at +4 °C for 16 h with 100 µL of diluted antibodies. Further analysis was performed as previously described [13]. Depending on the studied parameter, either polyclonal sheep antibodies against Lp(a) or LDL, conjugated with peroxidase (Figure 2C,D), were used as developing (second) antibodies. Studied samples were diluted ten times and titrated in 1:3 increments.

Statistical analysis of the results was conducted with MedCalc 15.8 software (MedCalc Software Ltd, Ostend, Belgium). Due to small numbers, non-parametric statistics were used. The relationships between different parameters were evaluated with Spearman rank correlation; comparison of independent samples was analyzed with the Mann–Whitney test. For comparison of related samples was carried out using the paired Wilcoxon test.

## 3. Results

**Figure 3:**
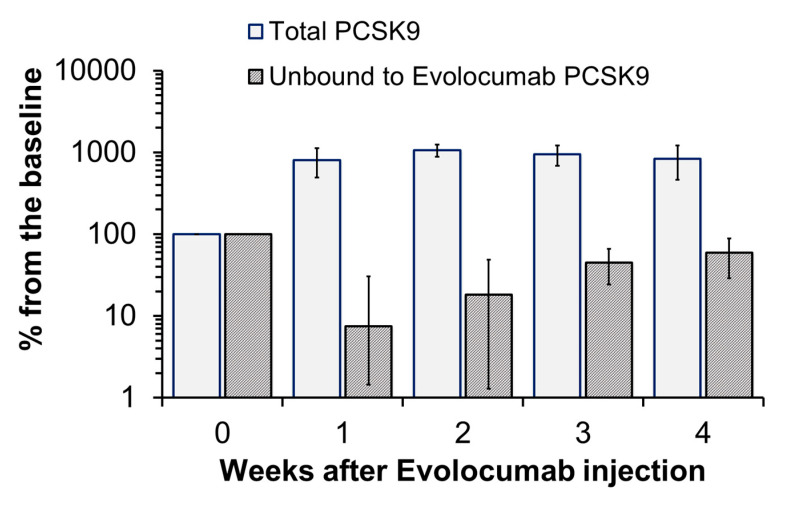
Changes in the concentrations of total PCSK9 and PCSK9 that is not associated with Evolocumab (% of the initial concentration taken as 100%).

After a single injection of 140 mg of MABs Evolocumab, the concentration of PCSK9 circulating in plasma increased in all participants from 496 [369; 719] to 3944 [3132; 4629] ng/mL (median [25; 75%]), and almost the entire pool of circulating PCSK9 remained associated with MABs for 2–3 weeks (Figure 3 and Table 1). Starting from the third week after the injection, the concentration of PCSK9 slowly decreased but did not reach its initial level in any of the subjects.

Furthermore, the level of LDL-C decreased significantly during the first two weeks after the injection from 3.36 mmol/L to 2.27 mmol/L. The level of TC reduced over the first week from 4.54 mmol/L to 3.30 mmol/L (Table 1).

**Figure 4:**
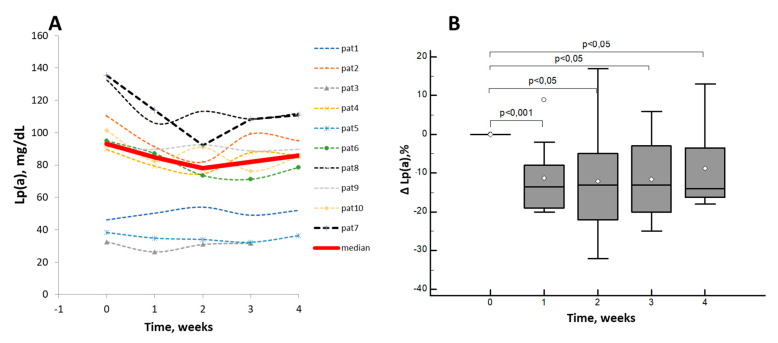
Changes in Lp(a) concentration after a single dose of Evolocumab: the concentration, mg/dL (**A**); percent changes from baseline (**B**). Data are presented as Box-and-Whisker plots, white dots indicate the mean values.

Lp(a) concentrations demonstrated multidirectional changes in different patients (Figure 4A) with the maximal decrease occurring on the second week after the drug administration. Despite the fact that the Lp(a) reduction was insignificant throughout the study, the median of changes in Lp(a) concentration varied from –13% [–24%; –2%] on the 2^nd^ week to –14% [–16%; –3%] at the last visit (Figure 4B).

There were no positive correlations between the changes of Lp(a) and LDL-C and TC, whereas there was a correlation between LDL-C and TC (r = 0.78, *p* < 0.0001) (Figures S1 and S2). The maximal decrease in LDL-C was –57%, the median on the 2nd week after the injection was –32% [–38%; –10%]. 

**Figure 5:**
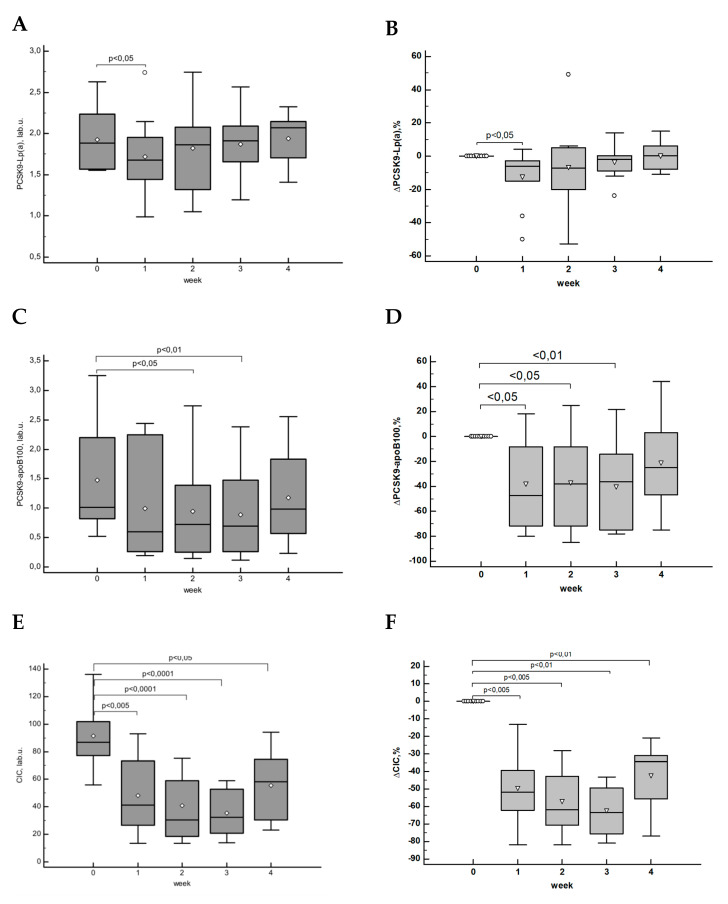
Absolute and percentage changes over time of circulating complexes of PCSK9–Lp(a) (**A**,**B**), PCSK9–apoB100 (**C**,**D**), and total pool of CIC (**E**,**F**).

The change in concentration of circulating complex of PCSK9–Lp(a) related to the dynamics of Lp(a) concentration was less marked, moreover, only the decrease during the 1^st^ week after the drug administration was significant according pair Wilcoxon test: median concentration of PCSK9-Lp(a) was 1.7 [1.3; 2.1] lab. u. after the 1^st^ week vs. 1,9 [1.6; 2.3] lab. u. before administration, the median of concentration changes was –5% [–8%; –2%] (Figure 5A,B).

On the contrary, the decrease in the level of circulating complexes of PCSK9–apoB100 was significant at the 2^nd^ week after the administration with the maximal reduction of –47% [–72%; –8%] on the 1^st^ week, and gradual increase (–38% [–72%; –8%]) from the 2^nd^ week (Figure 5C,D).

The changes in CIC level demonstrated a significant decrease related to baseline throughout the study; the maximal change was achieved at weeks 2 and 3 by –62% [–70%; –43%] and –63%[–75%; –50%], respectively (Figure 5E,F). According to the correlation analysis, there was no association between the changes in Lp(a) concentration and circulating complexes of PCSK9–apoB100 (r = 0.24, *p* > 0.05) and PCSK9–Lp(a) (r = 0.15, *p* > 0.05), while the correlation between the changes in levels of PCSK9–apoB100 complex and LDL-C was significant (r = 0.31, *p* < 0.05). We found a significant correlation between the dynamic of CIC and circulating complexes of PCSK9–apoB100 and PCSK9–Lp(a) (r = 0.36 and r = 0.33, *p* < 0.05 for both). In addition, the changes of CIC were not correlated with the changes in concentrations of IgG (r = 0.22, *p* = 0.15) or IgM (r = 0.10, *p* = 0.52). 

The dynamic of Lp(a) concentration was associated with changes of PCSK9-Lp(a) complex (r_parcial_ = 0.44, *p* < 0.05) after being adjusted to age, sex, time after MABs administration, changes of unbound PCSK9, CIC and PCSK9-apoB100 complex. The dynamics of LDL-C in a same model was associated with the changes of concentration of two complexes PCSK9-Lp(a) and PCSK9-apoB100 (r_parcial_ 0.45 and r_parcial_ 0.49, *p* < 0.05 for both). The dynamic of CIC was not an independent factor of Lp(a) or LDL-C decreasing in multiple regression.

## 4. Discussion

Increasing data suggest that the effect of PCSK9 on LDL cholesterol and on atherosclerosis in particular is not limited solely to its effect on the LDL receptor [14,15,16,17]. The ability of therapeutic MABs against PCSK9 to reduce the level of LDL-C in patients with homozygous FH and defective alleles of LDLR also demonstrates the possibility of a receptor-independent effect on LDL cholesterol [18].

The precise mechanisms of the dose-dependent decrease in Lp(a) concentration of up to 30% after therapy with PCSK9 inhibitors [6] are still not clear; their understanding may help to clarify details of Lp(a) metabolism [10]. Despite the similarity of the structural organization of Lp(a) and LDL particles and the presence of an apoB100 molecule in both, the LDL receptor was shown not to be important for the catabolism of Lp(a), in transgenic animals [19], cell models [20], clinically [7,21] and in kinetic studies [22]. Statins promote the increase in the number of LDL receptors on hepatocytes and result in an increase of Lp(a) concentration versus a decline of LDL-C [2]. However, it has been shown that Lp(a) internalization by hepatocyte cell lines increased both after the enhanced expression of LDL receptor and blocking of PCSK9 synthesis [23]. The effect of PCSK9 on the degradation of other receptors potentially involved in the catabolism of Lp(a) [24], such as LDL receptor-bound protein 1 [25] and scavenger receptor B1, can also reduce the Lp(a) concentration. However, it was demonstrated that Alirocumab did not affect Lp(a) uptake by hepatocytes and human skin fibroblasts [26]. Plasminogen receptors may also be involved in the Lp(a) degradation via the lysine-binding sites of apo(a) [20]. However, data on the possible effect of PCSK9 on them is limited only by annexin A2 [24]. Furthermore, the results of recent studies with primates demonstrated the influence of PCSK9 inhibitors on Lp(a) production but not on catabolism [27]. An alternative mechanism might involve the elimination of circulating immune complexes formed by therapeutic MABs and PCSK9 associated with apoB100–containing lipoproteins and Lp(a), in particular [28]. The presence of these complexes has been described in patients with CHD and elevated Lp(a) [29], FH [13] as well as in healthy volunteers [30]. The concentration of circulating PCSK9-Lp(a) complex it is relatively stable without medication treatment overtime according to our observations and the IONIS-APO(a)Rx phase II trial [31]. Most of the available studies on the mechanisms of the influence of PCSK9 inhibitors on the Lp(a) were carried out on cell cultures and animal models, so their results should be extrapolated to humans with great caution. FOURIER study with high-risk patients, was shown that the efficacy of Lp(a) reduction due to Evolocumab administration for 48 weeks depended on the initial Lp(a) concentration and was poorly correlated with changes in LDL-C concentrations [32]. The same results were seen in our study.

Our study is the first one to explore the influence of Evolocumab on the dynamics of circulating complexes of PCSK9 with apoB100–containing lipoproteins [PCSK9-Lp(a) and PCSK9-apoB] in humans. These results also interesting as a try to assess one of the possible mechanisms of decreasing Lp(a) through the removal of the MAB-PСSK9-Lp(a) triple complex from the blood. We discovered that a single administration of Evolocumab resulted in tenfold increase of PCSK9 concentration in blood serum from 496 [369; 719] ng/mL to 3944 [3132; 4629] ng/mL, with almost the whole pool of circulating PCSK9 being bound to MABs during the 2–3 weeks (Figure 3) following its injection. According to the existing data, it could be expected that recirculation of LDL receptors on the surface of hepatocytes would be elevated over two weeks and therefore internalization of Lp(a) could follow the same pathway. However, we observed a relatively high variability in Lp(a) dynamics, absence of correlation with changes in LDL-C and Lp(a)-corrected LDL-C (LDL-Ccorr), indicating the absence or limited effect of the LDL receptor on Lp(a) catabolism. Corresponding with the reduction in LDL-C concentration, we also found a significant decrease in the level of circulating complex of PCSK9–apoB100 of –48%, without changes in PSCK9–Lp(a) complex concentration (–5%). We suggest that a decrease in LDL-C concentration occurs both due to an increase in receptor-mediated uptake of LDL, and due to the activation of the complement system, which corresponds to a decrease in the total CIC after MABs administration. Formation of complex of PCSK9 with therapeutic antibodies was most prominent during the first 14 days after drug administration. The absence of a detectable amount of PCSK9 unbound to Evolocumab caused neither a significant change in PCSK9–Lp(a) complex, nor did it change the Lp(a) level. Therefore, elimination of MAB–PCSK9–Lp(a) triple complexes from the bloodstream cannot be the main mechanism of influencing on the Lp(a) level; however, changes in the content of such complexes were observed.

Limitations: This study was uncontrolled. However, the absence of control group could not be a confusing factor because we obtained the level of Lp(a) and its complex with PCSK9 before and after Evolocumab injection. Uncontrolled studies can be quite useful to find out whether a treatment causes any new effects or an effect on a new biomarker. We did not anticipate changes in PCSK9-Lp(a) complex concentrations without treatment because it is relatively stable over time in healthy subjects. So, these individuals could be served as self-controls.

## 5. Conclusions

A single administration of Evolocumab in healthy volunteers with elevated Lp(a) levels resulted in a 14% decrease of Lp(a) concentration, a 5% decrease in circulating complexes of PCSK9–Lp(a), a significant reduction of LDL-C concentration of 36%, a reduction in circulating complexes of PCSK9–apoB100 of 47%, and a tenfold increase in serum PCSK9 concentration. Elimination of circulating complexes of MAB–PCSK9–Lp(a) does not appear to be a significant explanation for the decrease in concentration of Lp(a) seen in subjects with elevated level of Lp(a) treated with PCSK9 monoclonal antibodies, in contrast to LDL.

## Figures and Tables

**Table 1:** Changes in serum lipids, Lp(a), unbound PCSK9, and circulating complexes of PCSK9 with lipoproteins and immunoglobulins during the 4 weeks after a single injection of Evolocumab 140 mg.

Parameter	Time (Week)				
	Baseline 0 Week	1^st^	2^nd^	3^rd^	4^th^
TC, mmol/L	4.54 [3.70;4.98]	3.30 [2.89;4.54]	3.77 [3.01;4,28] ^a^	3.92 [3.57;4.40]	4.45 [3.90;4.81]
LDL-C, mmol/L	3.47 [2,70;3,79]	2.47 [1.66;3.17] ^a^	2.27 [2.10;2.70] ^a^	2.23 [2.07;2.87] ^a^	2.75 [2.57;3.22]
LDL-Ccorr, mmol/L	2.60 [2,0;3,2]	1.95 [1.30;2.50] ^a^	1.70 [1.50;2.00] ^a^	1.90 [1.25;2.07]	2.30 [1.65; 2.48]
TG, mmol/L	0.84 [0.58;0.98]	0.72 [0.63;0.89]	0.78 [0.69;1.05]	0.73 [0.67;80.93]	0.90 [0.54;1.30]
HDL, mmol/L	0.99 [0.73;1.25]	1.03 [0.94; 1.45]	1.14 [0.91;1.19]	1.22 [1.10;1.29]	1.35 [0.79;1.93]
Lp(a), mg/dL	93.14 [46.10;110.40]	84.70 [50.20;91.30] ^a^	78.15 [54.00;92.10] ^a^	82.05 [49.00;99.30] ^a^	85.80 [71.78;99.00] ^a^
PCSK9, unbound, ng/mL	710 [520;1062]	0 [0;0] ^a^	0 [0;13] ^b^	450 [190;503] ^a^	341 [142;504] ^a^
Total pool of PCSK9, ng/mL	496 [369;719]	3944 [3132;4629] ^a^	5916 [3694;7332] ^a^	5175 [3621;5832] ^a^	3895 [3442;3964] ^a^
[PCSK9-apoB], lab.u.	1.01 [0.82;2.20]	0.60 [0.25;2.24]	0.73 [0.25;1.39] ^a^	0.69 [0.26;1.48] ^a^	0.98 [0.57;1.83]
[PCSK9-Lp(a)], lab.u.	1.89 [1.57;2.23]	1.68 [1.44;1.95] ^a^	1.86 [1.32;2.08]	1.91 [1.66;2.09]	2.07 [1.70;2.14]
IgG, g/L	10.83 [9.24;12.03]	9.93 [8.30;12.04]	9.71 [8.56;13.12]	10.36 [9.41;12.04]	9.62 [8.51;11.62]
IgM, g/L	0.83 [0.67;1.12]	0.76 [0.58;1.07]	0.64 [0.58;1.25]	0.68 [0.54;0.93]	0.59 [0.52;1.13]
CIC, lab.u	86.80 [77.05;102.00]	41.30 [26.45;73.22] ^b^	30.40 [18.37;59.03] ^b^	32.25 [20.70;52.75] ^a^	58.10 [30.50;74.60] ^a^

Data are presented as median [25%; 75%], ^a^ *p* < 0.05, ^b^ *p* < 0.005 regarding the level before the injection according to paired samples Wilcoxon test. Abbreviations: Lp(a)—lipoprotein(a), TC—total cholesterol, TG—triglycerides, HDL—high-density lipoproteins, PCSK9—proprotein convertase subtilisin/kexin type 9, CIC—circulating immune complexes, IgG—immunoglobulins G, IgM—immunoglobulins M, lab.u.—laboratory units.

## Supplementary Materials

The following are available online at https://www.mdpi.com/2308-3425/7/4/45/s1, Figure S1: Association of the changes of Lp(a) and TC with LDL-C after single Evolocumab administration. Figure S2: Association of the changes of Lp(a) with TC after single Evolocumab administration.
