# Supplementary material for: Effect of Evolocumab on Lipoprotein(a) and PCSK9 in Healthy Individuals with Elevated Lipoprotein(a) Level
Source: J Cardiovasc Dev Dis. 2020 Oct 15;7(4):45. doi: 10.3390/jcdd7040045 (PMC7712661; doi:10.3390/jcdd7040045)
Supplement: Supplementary file 1 [file jcdd-07-00045-s001.pdf]

**SUPPLEMENT MATERIALS**

# Effect of Evolocumab on Lipoprotein(a) and PCSK9 in healthy individuals with elevated Lipoprotein(a) level

O. Afanasieva, M. Ezhov, E. Klesareva, et al

National Medical Research Center of Cardiology, Ministry of Health of the Russian Federation, Moscow, Russia

***ELISA for determination of the level of PCSK9 bound with MABs and total PCSK9 (unbound and bound with MABs).***

The total amount of PCSK9 in serum was determined with a Human Proprotein Convertase 9/PCSK9 kit (R&D Systems, United States). To estimate free PCSK9 unbound from therapeutic antibodies in serum, the same R&D Systems kit was used with the following modification of the coupled antibodies on the plates: 100 µL of human MABs against PCSK9 with the concentration of 140 mg/dL (Evolocumab, AMGEN, Netherlands) were diluted in 10 mM phosphate buffer with pH of 7.4 and coupled in Nunc MaxiSorb plates (Denmark) at a final concentration of 100 µg/mL. These plates were used to determine the concentration of PCSK9 unbound from MABs. The calibrator, control serum, HRPO conjugated antibodies, and the sequence of the assay itself were completely identical to the assay for determination of the total amount of PCSK9 using the R&D kit (Suppl Fig. 1 A, B).

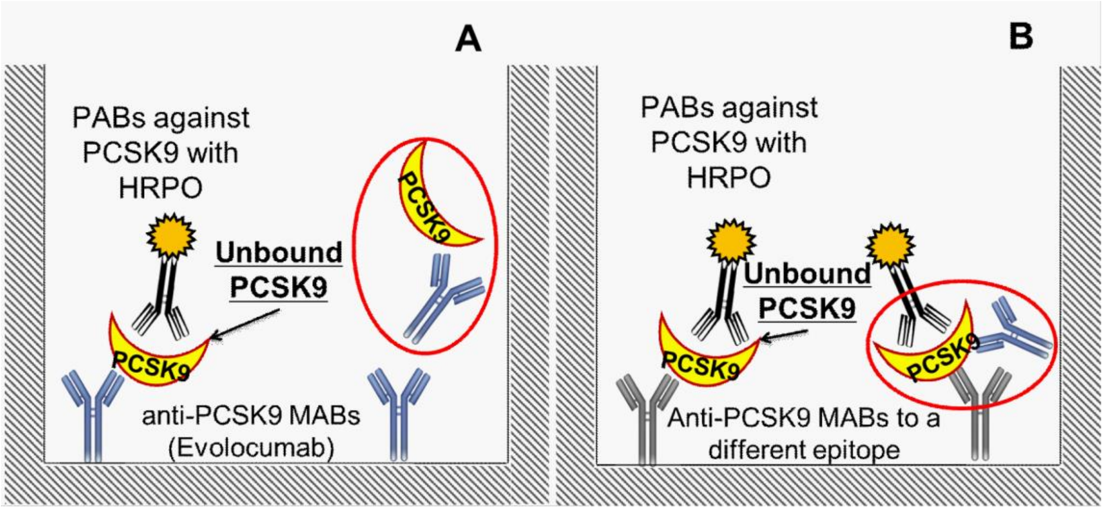


**Suppl Fig. 1.** ELISA scheme for measuring PCSK9 not associated with MABs (A) and total pool PCSK9 unbound from and bound with MABs (B). Red circles highlight the complex of PCSK9 with MABs (Evolocumab).

Abbreviations: PCSK9 - proprotein convertase type 9 subtilisin / kexin, MABs - monoclonal antibodies, PABs - polyclonal antibodies, HRPO - horseradish peroxidase, LDL - low density lipoproteins.

***ELISA for determination of complex PCSK9 and apoB100-containing lipoprotein determination*.**

To determine the concentrations of the complexes of PCSK9 with apoB100-containing lipoproteins: Lp(a)–PCSK9 and apoB100–PCSK9, samples of human MABs against PCSK9 with a concentration of 140 mg/dL (Evolocumab, AMGEN, Netherlands) were diluted in 10 mM phosphate buffer with pH of 7.4 to a final concentration of 100 μg/mL and ELISA plates (Costar, United States) were incubated for one hour at 37 ℃, then at +4 ℃ for 16 hours with 100 μL of diluted antibodies.

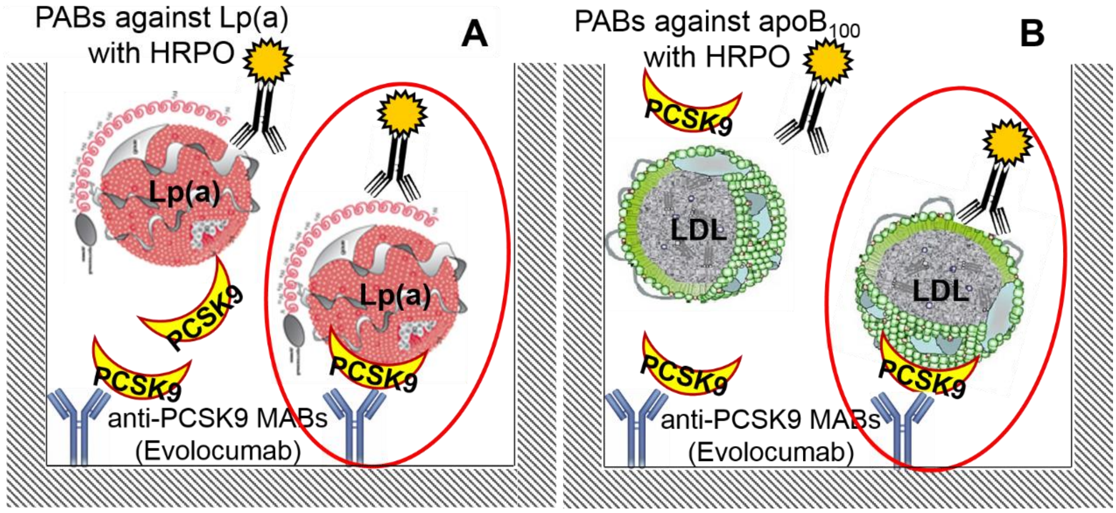


**Suppl Fig. 2.** ELISA scheme for measuring PCSK9-Lp(a) (A) and PCSK9-apoB100 complexes (B). Red circles are demonstrated the detectable complex of coupled antibodies-antigen-second antibodies conjugated with HRPO.

Abbreviations: Lp(a) - lipoprotein (a), PCSK9 - proprotein convertase type 9 subtilisin / kexin, MABs - monoclonal antibodies, PABs - polyclonal antibodies, HRPO - horseradish peroxidase, LDL - low density lipoproteins.

Association of  the changes of Lp(a) and TC with LDL-C after single Evolocumab administrations

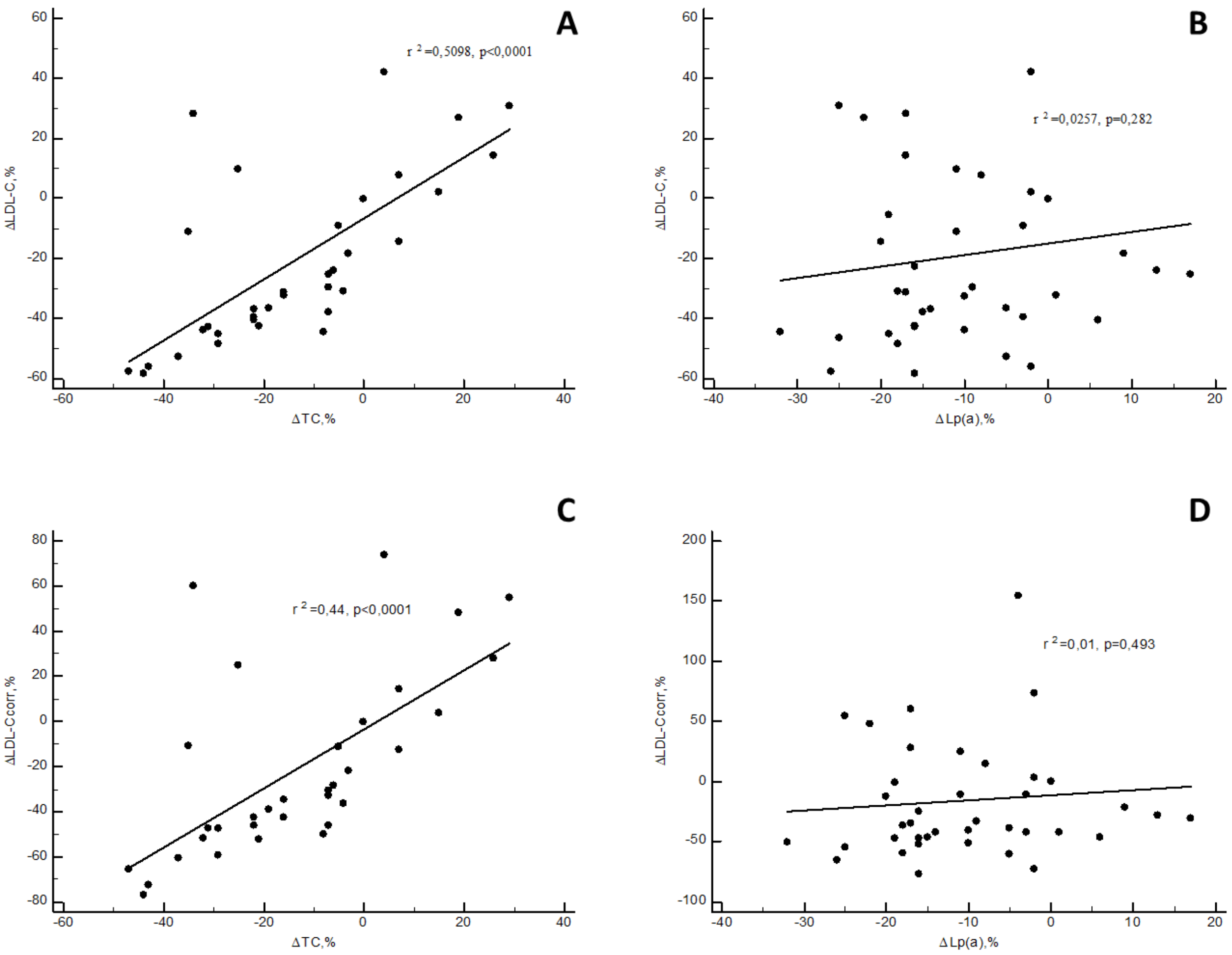


**Suppl Fig. 3.** The correlation between the changes in concentrations of LDL-C (A, B) and LDL–Ccorr (C, D) with concentrations of TC (A, C), and Lp(a) (B, D).

There were no positive correlations between the changes of Lp(a) and LDL-C, whereas there was a correlation between LDL-C and TC ($r=0.78$, $p<0.0001$).  This means that the decrease in total cholesterol occurs due to LDL-C, and not due to a decrease in Lp(a)-cholesterol
